# Guinea pig immunoglobulin VH and VL naïve repertoire analysis

**DOI:** 10.1371/journal.pone.0208977

**Published:** 2018-12-13

**Authors:** Shun Matsuzawa, Masaharu Isobe, Nobuyuki Kurosawa

**Affiliations:** 1 Laboratory of Molecular and Cellular Biology, Graduate School of Science and Engineering for Research, University of Toyama, Toyama-shi, Toyama, Japan; 2 Medical & Biological Laboratories Co., Ltd., Ina-shi, Nagano, Japan; Chang Gung University, TAIWAN

## Abstract

The guinea pig has been used as a model to study various human infectious diseases because of its similarity to humans regarding symptoms and immune response, but little is known about the humoral immune response. To better understand the mechanism underlying the generation of the antibody repertoire in guinea pigs, we performed deep sequencing of full-length immunoglobulin variable chains from naïve B and plasma cells. We gathered and analyzed nearly 16,000 full-length V_H_, V_κ_ and V_λ_ genes and analyzed V and J gene segment usage profiles and mutation statuses by annotating recently reported genome data of guinea pig immunoglobulin genes. We found that approximately 70% of heavy, 73% of kappa and 81% of lambda functional germline V gene segments are integrated into the actual V(D)J recombination events. We also found preferential use of a particular V gene segment and accumulated mutation in CDRs 1 and 2 in antigen-specific plasma cells. Our study represents the first attempt to characterize sequence diversity in the expressed guinea pig antibody repertoire and provides significant insight into antibody repertoire generation and Ig-based immunity of guinea pigs.

## Introduction

Immunoglobulins (Igs), a major component of the adaptive immune response, are heterodimeric proteins composed of two heavy (H) and two light (L) chains. Each Ig chain consists of variable (V) domains that have great genetic diversity and specifically bind antigen epitopes [1]. V gene diversity is generated by various strategies that differ in different species [2]. Primary V gene diversity in mice and humans is generated by V(D)J recombination, which rearranges multiple germline V, diversity (D) and junction (J) gene segments to the functional V gene [3]. During V(D)J recombination, junctional diversity is generated by recombination-associated nucleotide deletion as well as addition of a series of palindromic, 'P' nucleotides followed by the addition of random ‘N’ nucleotides [1]. The complementarity-determining region 3 of V_H_ (CDRH3) is the most diverse region because it is composed of V, D and J gene segments [3]. After V(D)J recombination, further V gene sequence diversity is produced through antigen-driven somatic hypermutation (SHM) [4]. In cattle and sheep, V(D)J combinatorial diversity is limited because only a few V genes rearrange [5, 6]. Therefore, they use SHM to generate their primary V gene diversity. After V(D)J recombination, cattle and sheep B cells migrate to ileal Peyer’s patches for additional diversification of the limited V(D)J combination by SHM [7, 8]. Cattle have exceptionally long CDRH3s, which have multiple cysteine residues mostly produced by SHM. The ultralong CDRH3s fold into a plethora of minidomains and create additional V gene diversity [9]. Furthermore, interior codons of D gene segments of the ultralong CDRH3s are deleted with high frequency, and their length and cysteine positions are altered to further diversify their sequences [10]. In chickens and rabbits, the combinatorial diversity is also limited. Therefore, they use gene conversionof homologous sequence from number of 5’ variable genes, which can be rearranged, and can donate diverse genetic fragments into the rearranged V domains [11–13].

The guinea pig (*Cavia porcellus*) is a species of rodent belonging to the family Caviidae. This animal shares similarity with humans with regard to hormonal and immunologic responses and has been used as an experimental model of various human infectious diseases, such as tuberculosis [14, 15], Zika virus infection [16] and Cytomegalovirus infection [17]. Guinea pigs have also been immunized for polyclonal antibody (pAb) production because they are phylogenetically distant from mice and rats. [18–22]. Recently, we developed a single cell-based monoclonal antibody (mAb) development method that enables guinea pig as a host animal for mAb production [23, 24]. Despite the importance of guinea pigs as laboratory and immunized animals, their immune system has not been well understood. Recently, Guo *et al*. characterized guinea pig Ig_H_ and Ig_L_ loci and found that the germline genetic components of the guinea pig antibody repertoire are large compared to those of humans, mice, and other vertebrates. Data from this study show that the Ig_H_ locus is composed of 507 V_H_ gene segments, including 94 potentially functional genes and 413 pseudogenes, 41 D_H_ gene segments, and six J_H_ gene segments; the Ig_κ_ locus is composed of 349 V_κ_ gene segments (111 potentially functional genes and 238 pseudogenes), three J_κ_ gene segments; and Ig_λ_ is composed of 142 V_λ_ gene segments (58 potentially functional genes and 84 pseudogenes) and 11 J_λ_ gene segments. The authors suggested that pseudogenes may serve as donor pools for gene conversion and contribute to Ig diversity in guinea pigs [25]. However, neither the gene usage in the guinea pig Ig repertoire nor the extent of nucleotide diversity from the germline sequence is known.

To address these questions, we amplified full-length V transcripts from naïve B cells obtained from spleens and lymph nodes from 2 guinea pigs and sequenced them using the 454 GS-FLX Plus next-generation sequencer (NGS). Our results indicate that the primary V gene diversity of guinea pigs is mainly produced via V(D)J recombination. Most of the germline V and J gene segments of the V_H_, V_κ_ and V_λ_ loci annotated as functional are integrated into the actual recombination events; therefore, combinatorial diversity is vast. We also compared V gene usage and mutation distribution between naïve B and antigen-specific plasma cells (PCs) and found that guinea pigs generate antibody diversity by both broader usage of V gene segments and an increased number of somatic mutations. Our study represents the first attempt to characterize sequence diversity in the expressed guinea pig antibody repertoire.

## Materials and methods

### Naïve guinea pig V gene library preparation and high-throughput sequencing

All animal studies were approved by the Committee for Laboratory Animal Care and Use at University of Toyama, and the experiments were carried out in accordance with approved guidelines (Protocol Number: A2016ENG-3). Guinea pigs purchased from Japan SLC, Inc. were immunized four times intramuscularly at the tail base with a 200 μL of 50:50 water-in-oil TiterMax Gold adjuvant emulsion containing 100 μg of antigens. Iliac lymph nodes and spleens were surgically removed from two individual naïve female Hartley guinea pigs under euthanasia with a barbiturate overdose. After the removal, the cells were dispersed, and B cells were stained with anti-guinea pig IgG (H + L) labeled with DyLight 650 (Abcam) followed by sorting with J-SAN cell sorter (BayBioscience). Approximately 2 x 10^5^ B cells were subjected to 5’-end homopolymer-tailed cDNA synthesis to amplify full-length V_H (μ)_, V_κ_ and V_λ_ genes, as described previously [23, 24]. The first round of PCR was performed with a universal 5’ RACE primer (Nhe polyC S) and a mixture of reverse primers specific for Ig constant region 1 (Gpig IgM AS1, Gpig IgK AS1 and Gpig IgL AS1). PCR was performed using PrimeStar DNA polymerase in 1 × PrimeStar GC buffer with the BIO-RAD MyCycler (35 cycles with denaturation at 98°C for 10 s, annealing and strand elongation at 55°C for 15 s and a final extension at 72°C for 60 s). The resulting PCR mixtures were diluted 1:8 with water, and 12 μL of each was used for the second round of PCR. The second round of PCR was performed with a forward primer designed for identifying each sample origin (Nhe-Eco47-S1, Nhe-Eco47-L1, Nhe-Eco47-S2 and Nhe-Eco47-L2) and a respective nested reverse primer (Gpig IgM AS2 FLX-Rv, Gpig IgK AS2 FLX-Rv, and Gpig IgL AS2 FLX-Rv). A unique 10-nt multiplex identifier (MID) for each sample origin was included in each forward primer between the adapter and the forward primer. The primers used are summarized in S1 Table. The PCR products were purified using a QIAquick PCR purification kit (Qiagen) and sequenced with 454 GS-FLX Plus NGS (Roche).

### Guinea pig IgBLAST database preparation

The potentially functional germline V and J gene segments of guinea pig Ig_H_, Ig_κ_ and Ig_λ_ annotated by Guo *et al*. [25] were obtained from the Broad Institute, which conducted genome sequencing and assembly (cavPor3, 6.79x coverage, Jul 2008). To achieve an accurate assignment of the V gene segment, we retrieved the V leader sequence and joined it in silico to its V gene segment to produce a custom guinea pig V database. The sequences are presented in the S1 File.

### Data preprocessing

All NGS data were first processed using the sequence quality of the 454 pipeline and filtered by nucleotide length >400 bp. The libraries were aligned to germline V gene segments in the custom databases described above, and aligned amino acid (aa) sequences containing stop codons were removed. The selected in-frame libraries were separated according to the MIDs for each sample origin. The preprocessed read sequences are presented in the S2 File.

### Ig gene analysis

The preprocessed reads were analyzed as follows:

- V and J gene segment combination frequency: Germline V and J gene segment assignments were derived from IgBLAST alignments against the custom IgBLAST database described above. V-J gene segment combination percent frequencies of all possible combinations were calculated. (ii) CDR3 length distribution: CDR3s were defined according to International ImMunoGeneTics (IMGT) unique numbering [26, 27]. Relative percent frequencies of CDR3 aa length of V_H_, V_κ_ and V_λ_ were calculated. (iii) Mutation frequency in the V gene segment: The number of nucleotide base substitutions, insertions and deletions from the aligned germline V gene segment sequence was counted to determine the number of mutations. Percent mutation frequencies were calculated by comparing the fraction of mutations in a given nucleotide to the fraction of that nucleotide in the germline sequences. Phylogenetic trees were compiled using Clustal X.

### Guinea pig monoclonal antibodies

The nucleotide sequences of guinea pig mAbs against CHK2 peptide, p53 peptide, RORγT peptide and human insulin prepared from antigen-specific PCs were used as described previously [23, 24].

## Results

### Data sets

**Fig 1:**
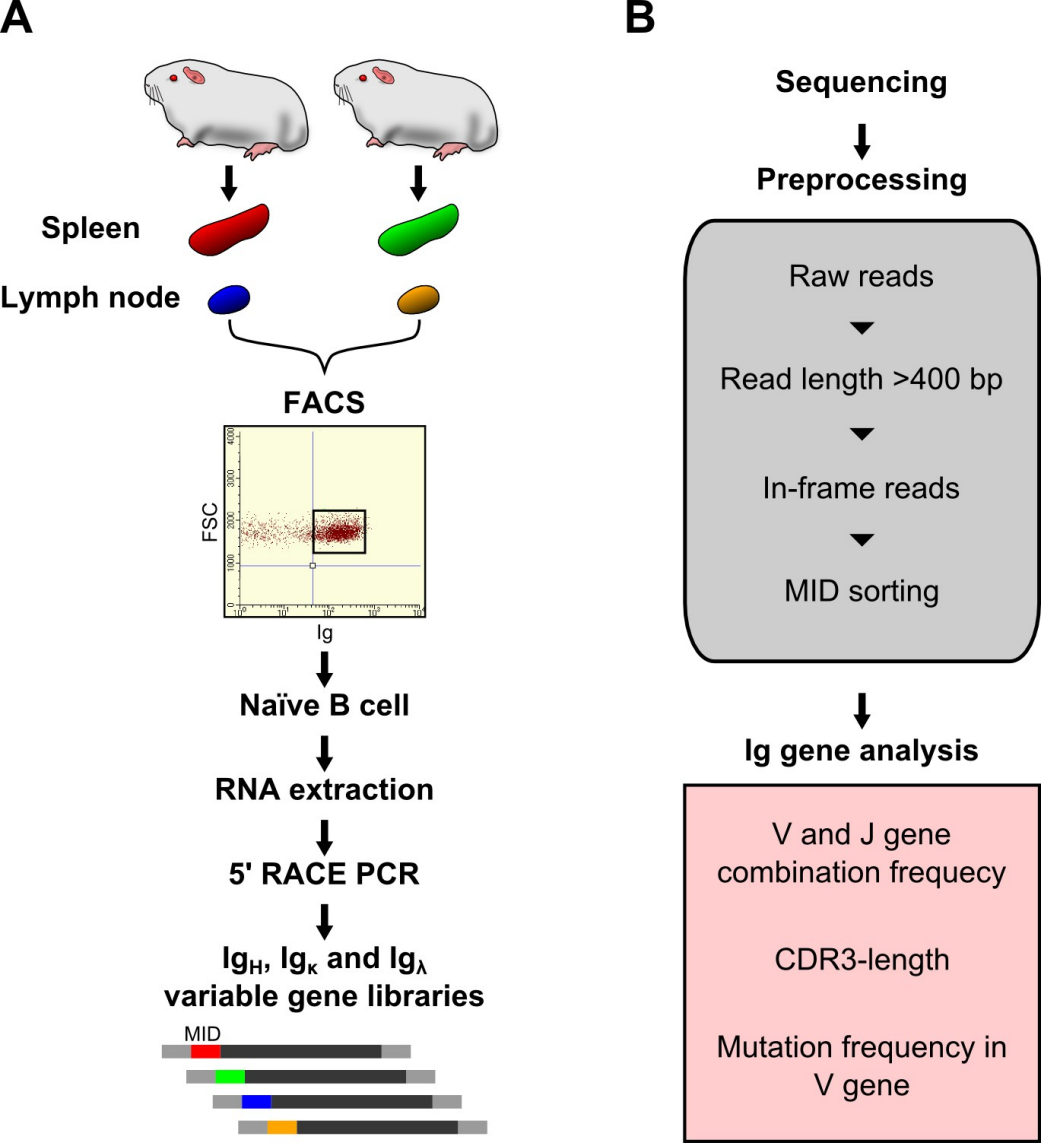
Workflow of NGS-based guinea pig naïve V gene analysis. (A) Naïve B cells in spleens and lymph nodes were isolated from two individual naïve guinea pigs by FACS. Each of the V_H(μ)_, V_κ_ and V_λ_ gene libraries was prepared by 5’ RACE PCR. Multiplex identifier (MID), which identifies the sample origin, is added with the sequencing adaptor. (B) The libraries are sequenced with the 454 GS-FLX Plus system. Raw data are first processed using the sequence quality, and in-frame reads with lengths greater than 400 bp are pooled. After partitioning each V(D)J combinatorial match into its own subset, IgBLAST alignments are performed using custom IgBLAST databases for guinea pigs. Output data are sorted by MID and analyzed for V gene usage frequency, CDR analysis, and mutation frequency.

To analyze antibody repertoires, we analyzed Ig transcripts from sorted B cell populations to obtain an overview of antibody repertoires in individual tissue. Full-length V gene transcripts of Ig_H_, Ig_κ_ and Ig_λ_ chains were amplified from B cell libraries by 5′ RACE PCR with universal forward primer containing unique 10-nt MID and reverse primers specific for the guinea pig Ig constant region of Ig_H(μ)_, Ig_κ_ and Ig_λ_. The amplified DNAs were sequenced by 454 pyrosequencing. The raw-read sequences were sorted into Ig_H_, Ig_κ_ and Ig_λ_ groups and checked for quality. Useful 42,771 Ig_H_, 60,308 Ig_κ_ and 45,643 Ig_λ_ sequencing reads were obtained (Table 1). Each group was then subjected to bioinformatics analysis as follows: (i) Length cutoff: sequences shorter than 400 nucleotide were removed; (ii) Translation: sequences containing stop codons were removed, and in-frame sequence containing homologies to conserved framework regions were collected; (iii) Custom database arrangement: Sequences containing productive V(D)J junctions were collected; (iv) MID sorting: Antibody repertoires between independent organs were analyzed (Fig 1). When we assigned each of the (i)- and (ii)-passed sequences to the best-scoring guinea pig germline V gene segment reference sequence, we found that 93.3% of transcribed V_H_ clone sequences, 83.9% of transcribed V_κ_ clone sequences and 95.9% of transcribed V_λ_ clone sequences showed more than 95% nucleotide identity with their germline sequences. These results suggest that gene conversion events are rare in rearranged guinea pig Ig diversification.

**Table 1:** Sequencing read counts from two individual naïve guinea pigs.

		V_H_	V_κ_	V_λ_
**Raw reads**		42771	60308	45643
**In-frame reads**		5675	9360	1230
**V identity >95%**	**Total**	5296	7852	1180
	**Spleen 1**	2388	1864	404
	**Lymph node 1**	526	1694	151
	**Spleen 2**	1366	2270	313
	**Lymph node 2**	1016	2024	312

### V and J gene segment usage in naïve guinea pig

**Fig 2:**
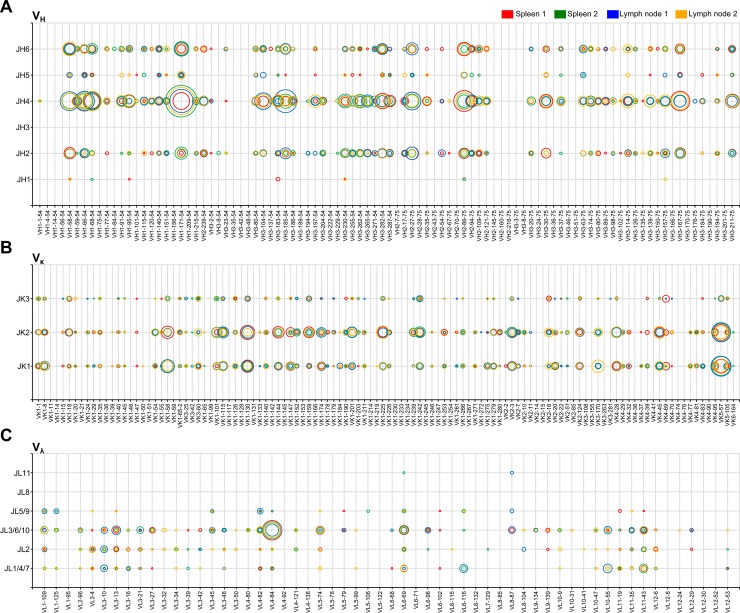
V and J gene segment usages in naïve B cells from individual guinea pigs. The x-axis represents the germline V gene segment, and the y-axis represents the germline J gene segment. Each point in the x-y axis represents all possible V-J gene segment combinations. The bubble size at each point corresponds to the percent frequency of reads from each sample matching a particular V-J gene segment combination. The bubble color indicates the sample origin, spleen (red) and lymph node (blue) from guinea pig no. one and spleen (green) and lymph node (orange) from guinea pig no. two.

We next analyzed V and J gene segment usage of transcribed V_H_, V_κ_ and V_λ_ genes in naïve B cells. The preprocessed reads were analyzed using a custom V gene database to assign the best-scoring germline V and J gene segment reference sequence, and the overall view of antibody repertoires in individual guinea pigs was visualized on 2D meshes in which the x-axis represented V gene segments, and the y-axis represented J gene segments (Fig 2).

There are 94 functional V_H_ and six functional J_H_ germline gene segments in a guinea pig, and our transcribed naïve Ig_H_ clone sequences were annotated to 66 (70.2%) of V_H_ and five (83.3%) of J_H_ gene segments, which represent 39.5% of the theoretical germline combination. The V_H_ usage appeared to have unequal frequencies, with ten V_H_ gene segments dominating 51.5% of the naïve Ig_H_ repertoire, in which VH1-171-54 (9.2%) was the most common V_H_ gene segment. The most common J_H_ gene segment was JH4 (64.0%); thus, the VH1-171-54 x JH4 combination shared 6.7% of the naïve Ig_H_ repertoire. JH3 was not used in the repertoire analyzed here.

There are 114 functional V_κ_ and three functional J_κ_ germline gene segments in a guinea pig, and our transcribed naïve Ig_κ_ clone sequences were annotated to 83 (72.8%) of V_κ_ and three (100%) of J_κ_ gene segments, which represent 63.7% of the theoretical germline combination. The V_κ_ usage also showed unequal frequencies, with ten V_κ_ gene segments dominating 49.6% of the naïve Ig_κ_ repertoire, in which VK5-57 (13.3%) was the most common V_κ_ gene segment. The most common J_κ_ gene segment was JK2 (50.8%); thus, the VK5-57 x JK2 combination shared 6.3% of the naïve Ig_κ_ repertoire.

It is interesting to note that guinea pigs use λ light chains, while κ light chains dominate over λ in mice [28]. There are 58 functional V_λ_ germline gene segments in a guinea pig, and our transcribed naïve Ig_λ_ clone sequences were annotated to 47 (81.0%) V_λ_ gene segments. Among the 11 functional J_λ_ germline gene segments, the nucleotide sequences of JL1, 4 and 7, JL3, 6 and 10, and JL5 and 9 are identical; therefore, we annotated our transcribed naïve Ig_λ_ clone sequences to six J_λ_ gene segment groups, including J1/4/7, J2, J3/6/10, J5/9, J8 and J11. Five out of six J_λ_ gene segment groups were used. The V_λ_ gene segment usage was also unequal, and the five V_λ_ gene segments dominated 52.8% of the entire naïve repertoire. The most common V_λ_ gene segment was VL4-84 (25.0%), and the most common J_λ_ segment was JL3/6/10 (62.0%). The VL4-84 x JL3/6/10 combination shared 23.7% of the naïve Ig_λ_ repertoire. No significant difference in V-J gene segment usage was observed in four MID samples, suggesting that each organ of two naïve guinea pigs performs a similar V(D)J recombination.

### CDR3 length distribution of V_H_, V_κ_ and V_λ_ genes in naïve guinea pig

**Fig 3:**
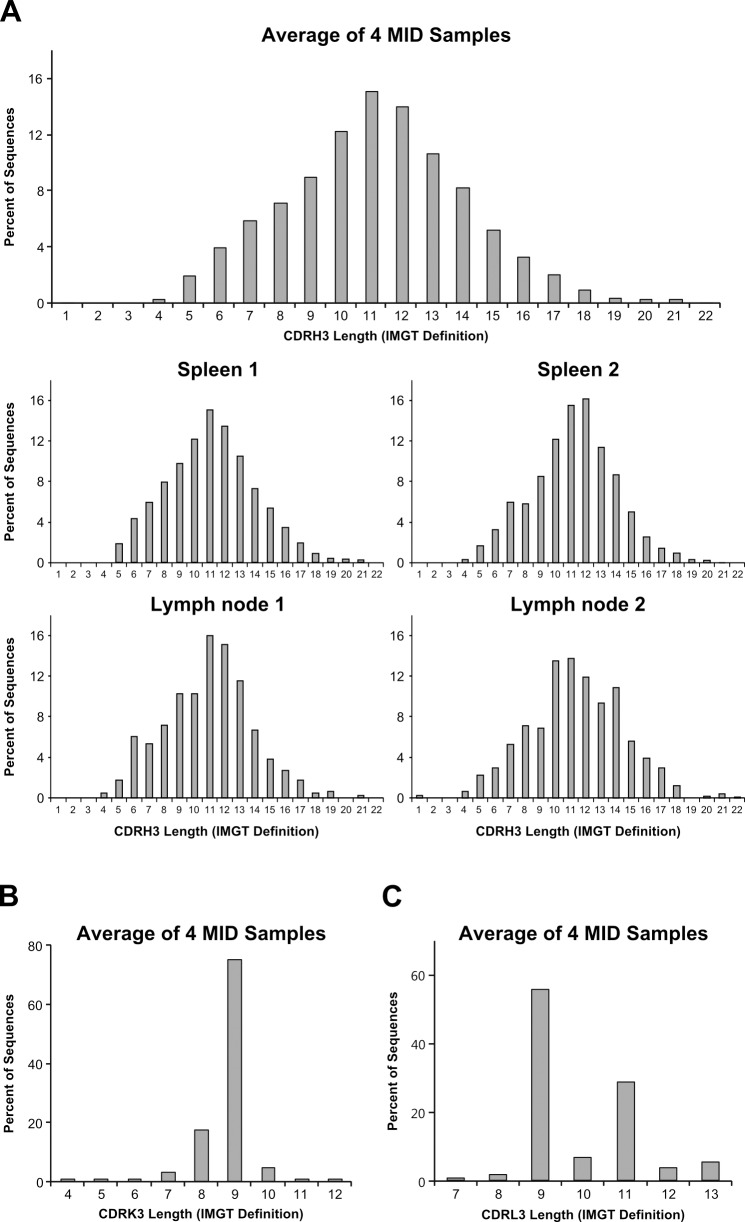
CDR3 length distributions of V_H_, V_κ_ and V_λ_ in naïve B cells. The relative frequencies of the CDR3 amino acid lengths of the V_H_ (A), V_κ_ (B) and V_λ_ (C) genes are shown. Data are from four MID samples (N = 5675 for V_H_, 9360 for V_κ_ and 1230 for V_λ_). CDR3s are defined according to IMGT unique numbering.

CDR3 is the result of V(D)J recombination and largely determines the diversity of the Ig repertoire; additionally, its length distribution differs among species [1, 29–32]. To understand the guinea pig Ig repertoire structure, we analyzed the naïve CDR3 length distributions of the V_H_, V_κ_ and V_λ_ genes (Fig 3 and S1 Fig). The V_H_ CDR3 length in guinea pigs ranged from five to 18 aa (98.9%), with the highest frequency at 11 aa (15.1%). Thus, the guinea pig V_H_ CDR3 length profile is similar to that of mice, which ranged from five to 26 aa with the highest frequency at ten aa [31]. V_κ_ CDR3 length in guinea pigs ranged from four to 12 aa with the highest frequency at nine aa (74.8%). V_λ_ CDR3 length in guinea pigs was not normally distributed, ranging from seven to 13 aa, with nine aa (55.2%) and 11 aa (28.4%) as frequently occurring lengths. The length distribution profile of naïve CDRs 1 and 2 of the V_H_, V_κ_ and V_λ_ genes closely mimicked those of the germline repertoire.

### Preferential use of a particular V gene segment with accumulated mutation in plasma cells

**Fig 4:**
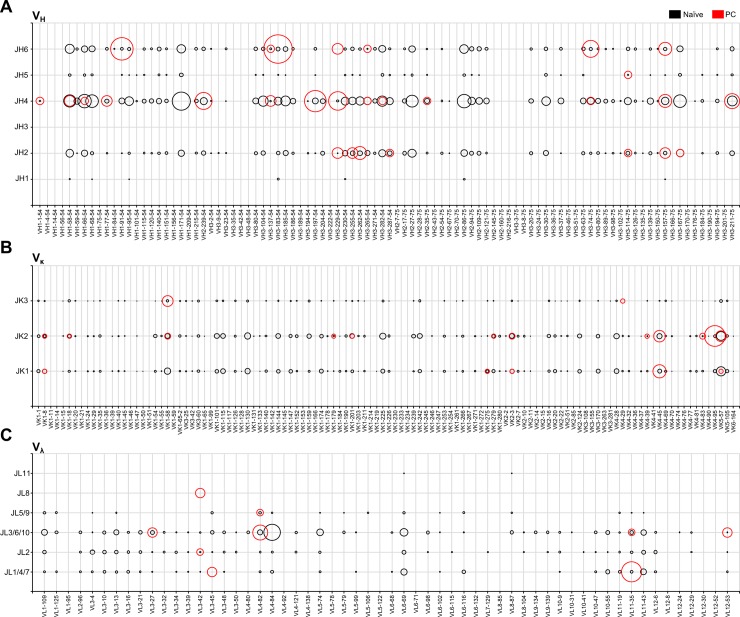
V and J gene segment usage in plasma cells from immunized guinea pigs. The x-axis represents the germline V gene segment, and the y-axis represents the germline J gene segment. Each point in the x-y axis represents all possible V-J gene segment combinations. The bubble size corresponds to the percent frequency of the V-J gene segment combination. Black bubbles represent total V and J gene segment usage in naïve B cells from four MID samples in Fig 2. Red bubbles represent the frequencies of V and J gene segment usage in plasma cells (N = 86 for V_H_, 68 for V_κ_ and 25 for V_λ_).

After antigen encounters, B celll clones with affinity to specific antigens undergo clonal selection and expansion, then terminally differentiated into PCs [1]. We previously isolated guinea pig mAbs against CHK2 peptide, p53 peptide, RORγT peptide and human insulin by directly cloning V genes from antigen-specific PCs. To understand the effect of immunization with foreign antigens on the guinea pig Ig repertoire, we sequenced 86 for V_H_, 68 for V_κ_, and 25 for V_λ_ genes from those mAb clones. Comparison of Ig repertoires between naïve B cells (Fig 4: red bubbles) and PCs (Fig 4: black bubbles) revealed striking bias in Ig_H_V and Ig_κ_V/Ig_λ_V gene usage in PCs. For example, the VH3-229-54 x JH6 gene segment combination appears to be frequently expressed in PCs, whereas the combination in naïve B cells is rare.

**Fig 5:**
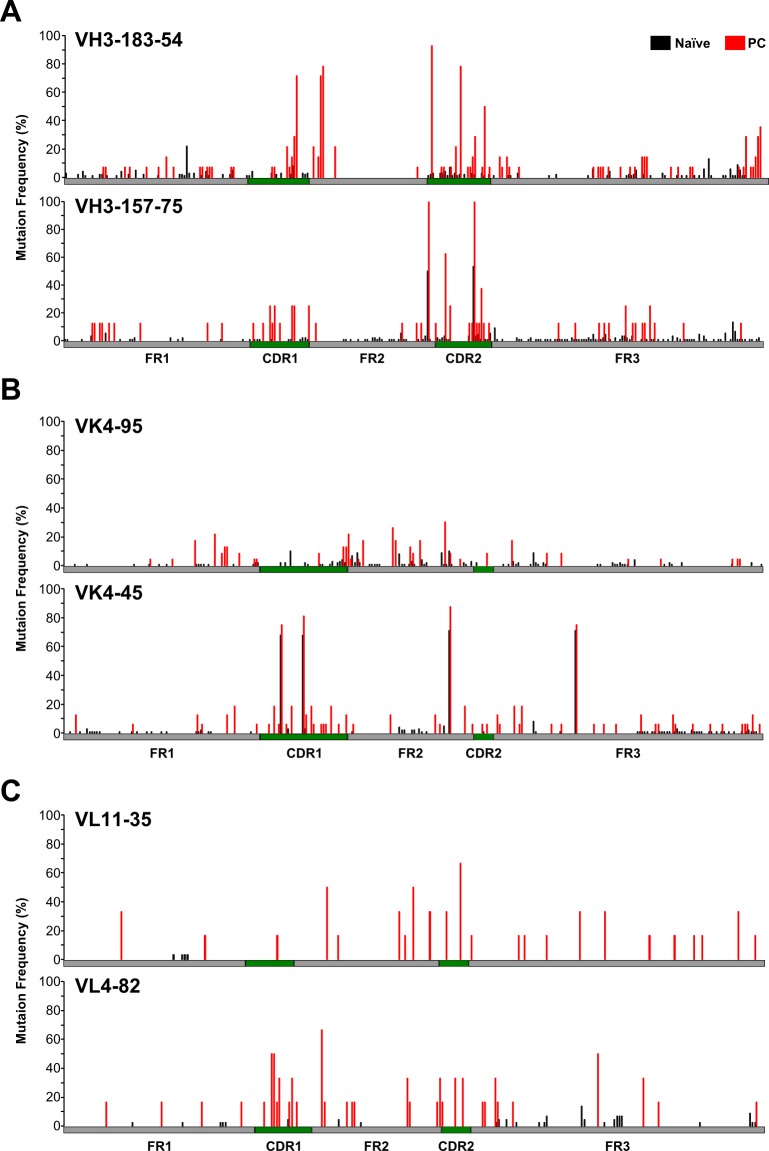
Distribution of mutational status in naïve and immunized guinea pigs. The x-axis represents nucleotide position defined according to IMGT unique numbering. The mutation frequencies in V gene segments (VH3-183-54, VH3-157-75, VK4-95, VK4-45, VL11-35 and VL4-82) of B cells in naïve guinea pigs (black) and plasma cells in immunized guinea pigs (red) are determined. Analyzed Ig sequence counts: VH3-183-54, naïve = 100, PC = 14; VH3-157-75, naïve = 88, PC = 8; VK4-95, naïve = 100, PC = 23; VK4-45, naïve = 100, PC = 16; VL11-35, naïve = 30, PC = 10; VL4-82, naïve = 44, PC = 6.

Next, we selected V gene segments that were expressed in both naïve B and PCs: VH3-183-54 (naïve = 100 and PC = 14), VH3-157-75 (naïve = 88 and PC = 8), VK4-95 (naïve = 100 and PC = 23), VK4-45 (naïve = 100 and PC = 16), VL11-35 (naïve = 30 and PC = 10), and VL4-82 (naïve = 44 and PC = 6). The selected reads were aligned to their germline V gene segments, and the relative mutation frequency was plotted on each germline V gene segment nucleotide sequence (Fig 5). We found higher mutations in all PC repertoires than in naïve B repertoires, which preferentially accumulated in CDRs 1 and 2 (Table 2). The higher mutation frequency may thus have been involved in the selection of the cells producing high affinity antibodies. Phylogenetic analysis of mAb clones of PCs derived from insulin-immunized guinea pigs (VH3-183-54 x JH6) revealed that these clones originate from the expansion and somatic hypermutation of a single B cell encoding an unmutated VH3-183-54 x JH6 antibody (S2 Fig).

**Table 2:** Mutation frequencies in naïve and immunized guinea pigs.

		Mutation Frequency (%)			
		FR	CDR	V	CDR/FR Ratio
**VH3-183-54**	**naïve**	0.7	1.3	0.8	1.8
	**PC**	3.0	8.3	3.9	2.8
**VH3-157-75**	**naïve**	1.1	1.9	1.2	1.8
	**PC**	1.9	10.2	3.3	5.3
**VK4-95**	**naïve**	0.6	0.8	0.6	1.4
	**PC**	1.4	1.0	1.3	0.7
**VK4-45**	**naïve**	1.0	3.2	1.4	3.2
	**PC**	2.1	7.1	2.9	3.4
**VL11-35**	**naïve**	0.1	0.0	0.1	0.0
	**PC**	1.3	2.6	1.5	1.9
**VL4-82**	**naïve**	0.4	0.1	0.4	0.2
	**PC**	2.0	8.3	3.0	4.1

FR, framework region; CDR, complementarity-determining region; V, V gene segment; PC, plasma cell

## Discussion

In this study, we analyzed the expressed B cell receptor repertoire of splenic and lymph node naïve B cells that were isolated from two naïve guinea pigs. We found that primary repertoire diversity is developed mainly via V(D)J recombination using a large number of functional Ig gene segments, and additional diversity is introduced with SHM.

Guinea pigs have relatively large potentially functional germline Ig gene segments in mammals that may greatly contribute to antibody diversity [5, 6, 25, 33]. Our V and J gene segment usage data of naïve guinea pig V_H_, V_κ_ and V_λ_ genes showed that most segments were used for actual V(D)J recombination events, indicating that the V(D)J combinatorial diversity in naïve guinea pigs is large.

Two Ig light chains, κ and λ, are used in mammalian species, and the relative abundance of κ and λ light chains varies among species: κ light chains dominate over λ in mouse, but λ light chains dominate over κ in cow and horse, whereas human and pig use both of them almost evenly [28]. It is worth noting that guinea pigs have 58 functional V_λ_ gene segments, and approximately 81.0% of them are rearranged with J_λ_ genes to express functional light chains. This feature may contribute to greater Ig_H_ and Ig_L_ combinatorial diversity in guinea pigs than in other rodents.

The number of VH genes and their subgroup composition varies by species, but placental mammalian VH segments can be classified into three clusters, clans I, II and III [34, 35]. Several species express only one or several closely related VH subgroups belong to either clan II (horse and cattle) or clan III (rabbit, dog, and pig) [32, 36–40]. Contrary, VH repertoires expressed in the mouse and human are distributed across all three mammalian VH clans, in which 22% of 181 functional VH segments are expressed in mouse, and most of 36–49 functional VH segments are expressed in human [41]. Guinea pig VH gene segments are divided into three subgroups (families 1, 2 and 3), in which families 1 and 2 belong to clan II, and family 3 belongs to clan III [25]. We have shown that at least 13 out of 22 functional VH genes (59%) in family 1, 10 out of 17 functional VH genes (58%) in family 2, and 35 out of 55 functional VH genes in family 3 were expressed. Taking the large number of functional VH genes and their high usage into account, expressed VH repertoire in guinea pig is larger than those found in mouse and human.

Another distinctive feature of the guinea pig germline V_H_ repertoire is that the number of pseudo-V_H_ gene segments is four times more than the potentially functional V_H_ gene segments. It is not known whether these pseudo-V gene segments serve as donor pools for gene conversion and contribute to Ig diversity in guinea pigs. In this study, we did not find clear evidence of a gene conversion event; however, it might be detected among Ig genes with a favorable arrangement of V genes and an antigenic stimulation that selects cells with conversions [42–44]. Because our data depth was not sufficient to cover the relatively rare repertoire, deeper read depth is needed to provide precise determination of naïve and antigen-stimulated guinea pig Ig repertoire structure and size.

Taking advantage of the evolutionary distance of guinea pigs between mice and humans, guinea pigs have been used as host animals for pAb production [18–22]. We recently developed a method for generating mAb from guinea pigs and showed that this animal can produce high affinity and specificity antibodies [23, 24]. To understand the effect of immunization with foreign antigens on guinea pig Ig repertoire, we analyzed V and J gene segment usage and mutation state of V_H_, V_κ_ and V_λ_ genes of PCs extracted from immunized guinea pigs. The V and J gene segment usage of the PCs suggested that immunization skewed the usage, and even relatively rare V-J gene segment combinations could be selected and amplified. We also found higher mutation frequencies in all PC repertoires with preferential accumulation in CDRs 1 and 2. These results imply a high frequency and stepwise occurrence of somatic point mutations in the expressed V genes and substantial clonal expansion of B cells in the guinea pigs and support the notion that guinea pigs can be a promising animal for antibody production. It has been shown that V-segment usage is influenced by a variety of factors including genetics, age and environment [45–47]. Further studies will be needed to understand the mechanism underlining the variance in V-segment usage in guinea pig.

Our study represents the first attempt to characterize sequence diversity in the expressed guinea pig antibody repertoire. These results provide significant insight into antibody repertoire generation and Ig-based immunity of guinea pigs.

## Supporting information

S1 Table The primers used in this study. (TIF)

S1 File The sequences used in the custom guinea pig IgBLAST database. (XLSX)

S2 File The NGS read sequences obtained in this study. (XLSX)

S1 Fig CDR3 length distributions of V_κ_ and V_λ_ in naïve guinea pig B cells. The relative frequencies of CDR3 amino acid length of V_κ_ (A) and V_λ_ (B) of each four MID samples are shown.
(TIF)

S2 Fig Phylogenetic tree of guinea pig naïve and immunized V_H_ genes of VH3-183-54 x JH6 gene segment combination. FR1 to FR4 nucleotide sequences of V_H_ genes were arranged in a phylogenetic tree denoting sequence similarity. Sequences of mAbs derived from insulin-immunized guinea pigs are shown in bold red. Sequences derived from naïve guinea pigs are shown in black.
(TIF)
