# Supplementary figures and images for: Guinea pig immunoglobulin VH and VL naïve repertoire analysis
Source: PLoS One. 2018 Dec 13;13(12):e0208977. doi: 10.1371/journal.pone.0208977 (PMC6292586; doi:10.1371/journal.pone.0208977)

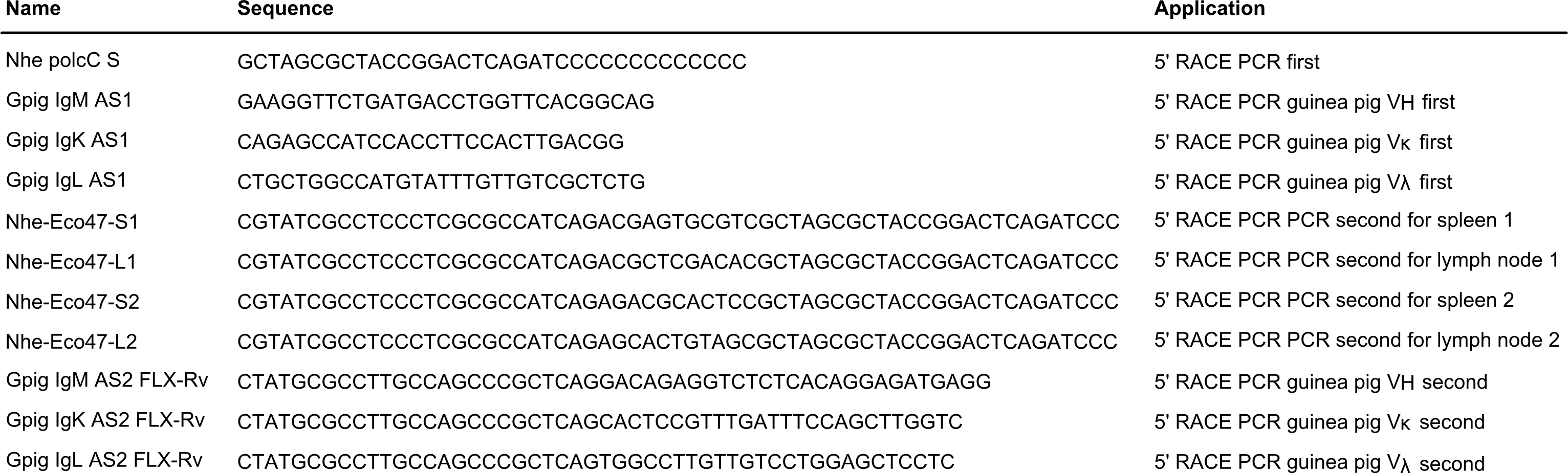

Supplement: S1 Table — (TIF) [file pone.0208977.s001.tif]

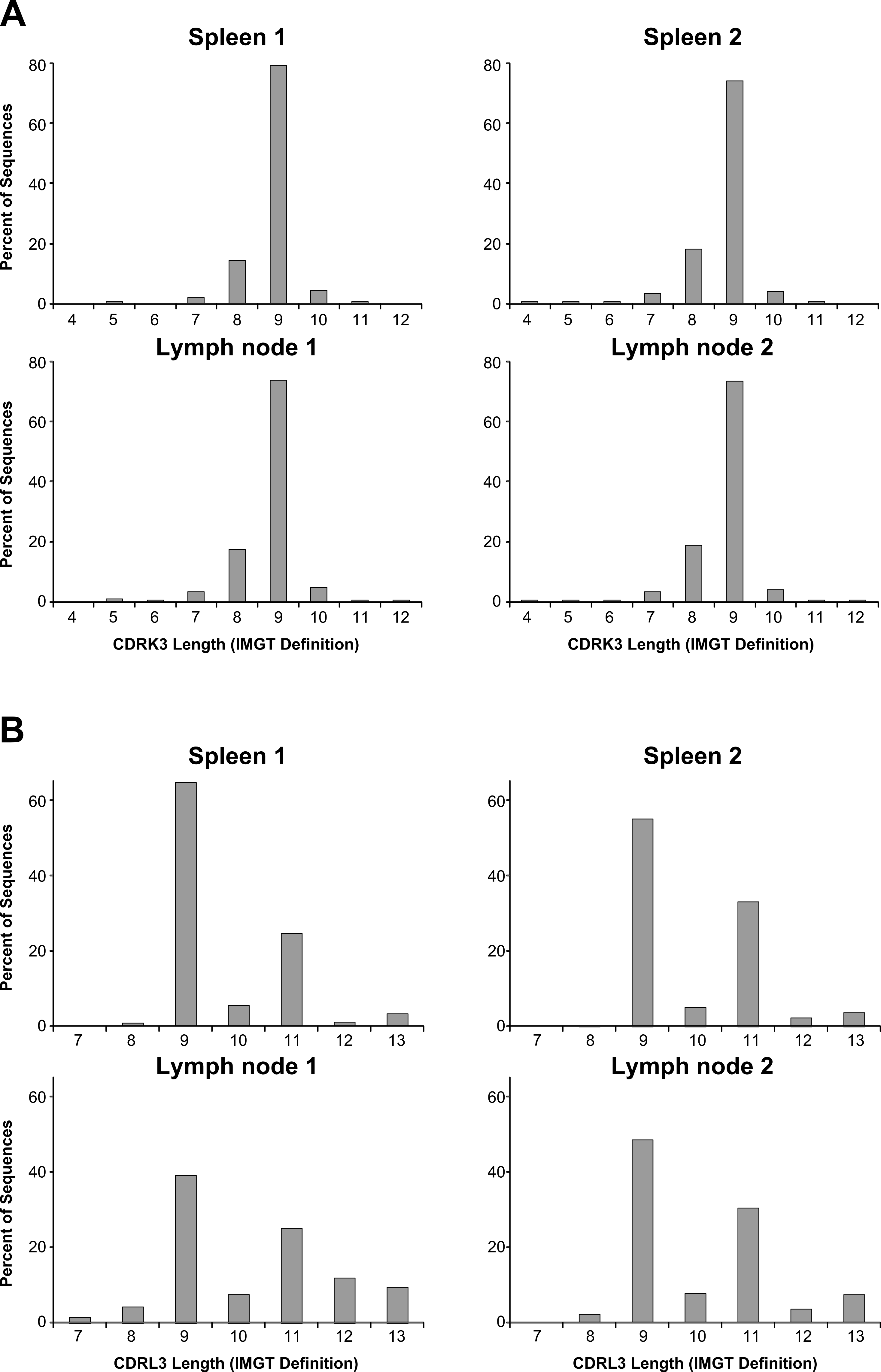

Supplement: S1 Fig — The relative frequencies of CDR3 amino acid length of Vκ (A) and Vλ (B) of each four MID samples are shown. (TIF) [file pone.0208977.s004.tif]

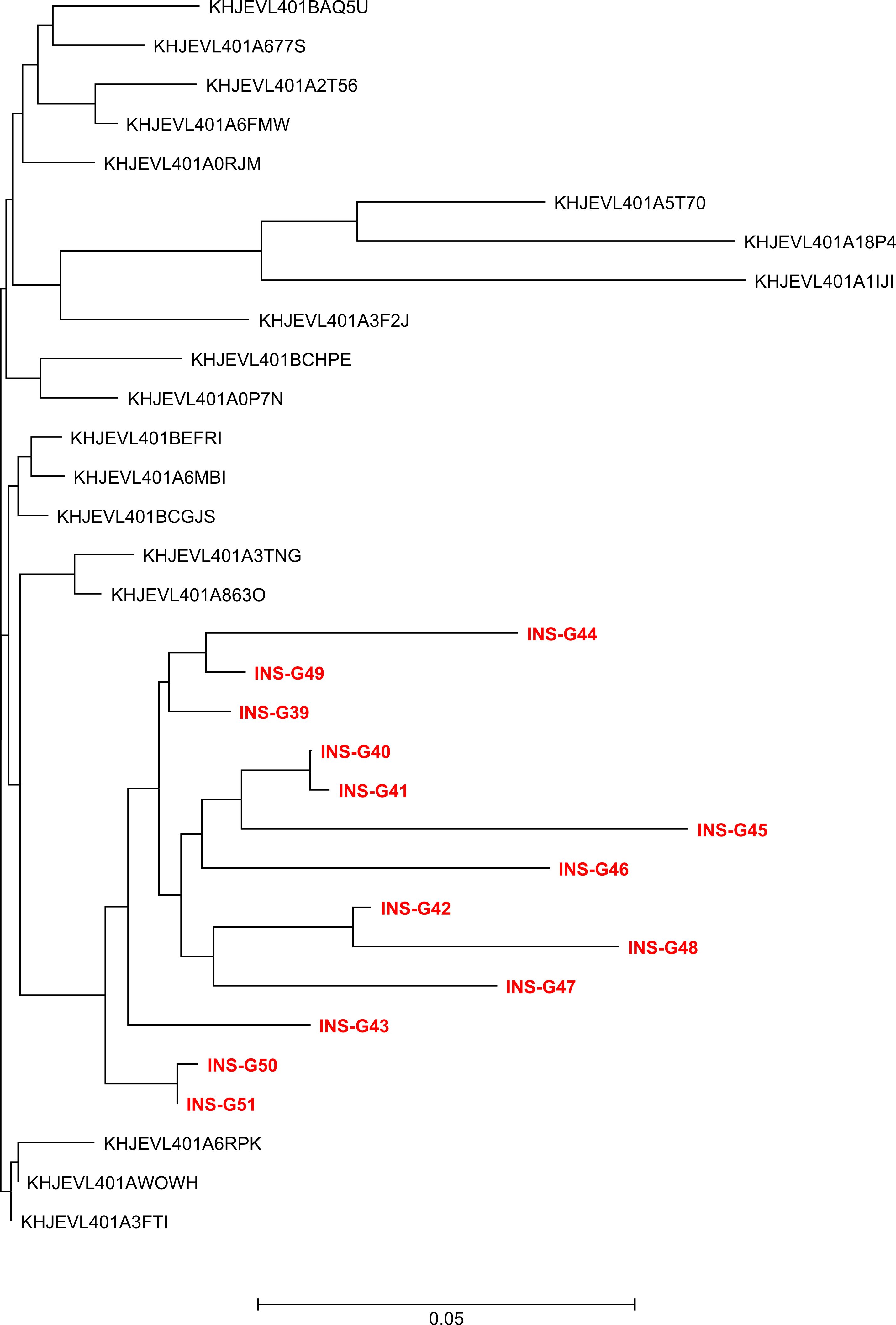

Supplement: S2 Fig — FR1 to FR4 nucleotide sequences of VH genes were arranged in a phylogenetic tree denoting sequence similarity. Sequences of mAbs derived from insulin-immunized guinea pigs are shown in bold red. Sequences derived from naïve guinea pigs are shown in black. (TIF) [file pone.0208977.s005.tif]
